# A nomogram based on baseline predictors for Graves’ disease relapse after antithyroid drug withdrawal: a retrospective study

**DOI:** 10.3389/fendo.2026.1968560

**Published:** 2026-10-22

**Authors:** Ruohan Dai, Mingrui Li, Yaohua Chen, Jiasi Wang, Hongquan Xiong

**Affiliations:** 1 Department of Endocrinology and Metabolism, Dazhou Central Hospital, Dazhou, China; 2 Department of Pulmonary and Critical Care Medicine, Dazhou Central Hospital, Dazhou, China; 3 Clinical Medicine Research Center, Dazhou Central Hospital, Dazhou, China; 4 Department of Otolaryngology-Head and Neck Surgery, Dazhou Central Hospital, Dazhou, China

**Keywords:** antithyroid drugs, Graves’ disease, GREAT score, nomogram, relapse, risk prediction

## Abstract

**Objective:**

To develop and validate a practical prediction model for relapse of Graves’ disease within one year after discontinuation of antithyroid drugs, using routinely available baseline characteristics, and to compare its performance with an existing score.

**Methods:**

This single-center retrospective cohort study enrolled 654 adults with Graves’ disease who achieved remission after at least 12 months of antithyroid drug therapy and subsequently discontinued treatment. The cohort was randomly split into training (70%) and testing (30%) sets. Predictors were selected by least absolute shrinkage and selection operator (LASSO) regression, and a multivariable logistic regression model was built on multiply imputed data. Model performance was assessed by discrimination, calibration, and decision curve analysis and was benchmarked against the Graves Recurrent Events After Therapy (GREAT) score. A nomogram was constructed to visualize individualized risk.

**Results:**

Five independent baseline predictors emerged: younger age, male sex, goiter grade 2–3, higher free thyroxine, and higher thyrotropin receptor antibody. The model demonstrated good discrimination, with an area under the receiver operating characteristic curve of 0.84 in the testing set, which showed discrimination comparable to that of the GREAT score (0.82; DeLong P = 0.31). Across quartiles of predicted probability, the observed relapse rate increased from 23.4% to 97.9% (test for trend p < 0.001). Calibration was satisfactory, and decision curve analysis indicated net clinical benefit across relevant threshold probabilities. The nomogram translates these five variables into a single probability of relapse. An interactive online calculator was concurrently developed to facilitate clinical use.

**Conclusions:**

Readily accessible clinical and laboratory parameters can identify patients at elevated risk of early relapse after antithyroid drug withdrawal for Graves’ disease. The proposed nomogram offers a simple, quantitative tool to support personalized treatment discussions and shared decision-making, although external validation in diverse populations is necessary before clinical implementation.

## 1 Introduction

Hyperthyroidism is a common endocrine disorder, affecting approximately 1.2% of the global population (1, 2). It is characterized by excessive secretion of thyroid hormones, which leads to a spectrum of clinical manifestations such as a systemic increase in metabolic rate (3, 4). The etiology of hyperthyroidism includes Graves’ disease (GD), toxic thyroid nodules, and thyroiditis, among which GD is the most prevalent subtype, accounting for 70% to 85% of cases (5, 6). Typical clinical features of GD encompass signs of systemic hypermetabolism, including weight loss, palpitations, and tremor, often accompanied by diffuse goiter and orbital manifestations such as exophthalmos (7).

With rapid socioeconomic development and growing health awareness in China, increasing attention has been directed toward the diagnosis and management of GD. Available therapeutic options include antithyroid drugs (ATDs), surgery, and radioactive iodine (RAI) therapy (8–10). Although thyroidectomy and RAI therapy are associated with lower relapse rates, they carry a substantial risk of permanent hypothyroidism, necessitating lifelong thyroid hormone replacement. In contrast, ATD therapy is cost-effective and less likely to result in permanent hypothyroidism, and therefore remains the first-line treatment for GD (11). ATDs act primarily by inhibiting the synthesis and release of thyroid hormones, thereby alleviating the symptoms of GD. However, the therapeutic effect of ATDs is often not durable, and the relapse rate is relatively high. Studies have shown that the relapse rate of GD after ATD withdrawal reaches 52% to 53% (12). This high relapse rate has been attributed to the gradual attenuation of ATD efficacy, interindividual variability, and drug-related adverse effects, resulting in the reappearance of symptoms shortly after treatment discontinuation in a considerable proportion of patients. Consequently, accurately predicting the risk of relapse after ATD therapy and formulating corresponding strategies for timely intervention to minimize relapse have emerged as a major clinical challenge.

Currently, the accuracy of prognostic prediction for GD in clinical practice remains suboptimal, and the lack of precise predictive tools contributes to difficulties in clinical decision-making. Existing prognostic assessment methods are predominantly based on simple clinical indicators and empirical judgment. For example, conventional predictors such as thyroid hormone levels and thyroid-related antibodies offer limited performance in individualized and precise risk prediction, making it difficult to deliver personalized management strategies. Moreover, most existing predictive models for GD relapse are data-driven, constrained by small sample sizes, poor generalizability, and a limited number of incorporated variables, and they inadequately integrate models with individual patient profiles (13–16). The strength of this study lies in its grounding in evidence-based medicine and real-world retrospective cohort data, focusing on the clinically challenging issue of relapse risk following ATD therapy for GD. By combining LASSO regression with multivariable logistic regression, we constructed a nomogram model with favorable predictive performance and clinical interpretability. This model can be applied to individualized risk assessment of relapse after drug withdrawal and serves as a quantitative tool for screening high-risk populations, facilitating risk communication, guiding follow-up management, and optimizing decisions regarding treatment discontinuation, thereby providing a basis for the refined management of GD.

## 2 Methods

### 2.1 Study design

This investigation is a single-center, retrospective cohort study. The cohort comprised patients newly diagnosed with GD who received ATDs as initial therapy at the Department of Endocrinology, Dazhou Central Hospital, between September 2019 and August 2025. The study population comprises patients with GD who achieved remission after at least 12 months of ATD therapy and subsequently discontinued treatment. All patients fulfilled the diagnostic criteria established by the Chinese guidelines for the diagnosis and management of hyperthyroidism. The study protocol was reviewed and approved by the Ethics Committee of Dazhou Central Hospital (Approval No. 2025-094). The requirement for informed consent was waived by the Ethics Committee of Dazhou Central Hospital due to the retrospective nature of the study. The inclusion criteria were as follows: (1) age 18 years or older; (2) an initial diagnosis of hyperthyroidism caused by GD, confirmed by clinical presentation, suppressed thyroid-stimulating hormone (TSH) levels accompanied by elevated free thyroxine (FT4) and/or free triiodothyronine (FT3), positive thyrotropin receptor antibody (TRAb), and imaging findings consistent with GD; (3) receipt of ATDs (methimazole or propylthiouracil) as the initial treatment, followed by achievement of remission and subsequent ATD withdrawal (as defined below); and (4) complete baseline data and a follow-up period of at least one year after ATD discontinuation. The exclusion criteria were as follows: (1) a diagnosis of thyroid malignancy, or receipt of thyroidectomy or radioactive iodine therapy before ATD withdrawal (i.e., as initial treatment or during the ATD treatment course, rather than as treatment for relapse occurring after discontinuation); (2) patients who were pregnant or lactating; (3) the presence of severe cardiac, hepatic, or renal insufficiency, or other major diseases potentially affecting follow-up or prognosis; (4) missing critical data that could not be imputed; and (5) patients lost to follow-up or whose outcomes could not be determined.

### 2.2 Remission

Remission was defined as the restoration and stable maintenance of normal thyroid function (TSH, FT3, and FT4 within their reference ranges) on a minimal maintenance dose of ATD after at least 12 months of standard therapy. ATD withdrawal was undertaken only after remission had been achieved and biochemical stability confirmed. The requirement for a minimum 12-month treatment course and confirmed remission before withdrawal defines the study population as patients who achieved remission on ATD therapy and subsequently discontinued treatment.

### 2.3 Data collection and definitions

Comprehensive baseline demographic, clinical, and laboratory data were collected for all eligible patients. Goiter grade (0–III) was assessed by the attending outpatient physician on physical examination according to standard criteria; grading was not performed by dual independent raters, and inter-observer variability was therefore not assessed. Candidate predictor variables included age at diagnosis, sex, time from symptom onset to the first clinic visit (in weeks), smoking history, the presence of Graves ophthalmopathy (GO), goiter grade (classified from 0 to III based on physical examination according to standard criteria), thyroid volume measured by ultrasound, baseline serum levels of TSH, FT3, FT4, TRAb, anti-thyroid peroxidase antibody (TPOAb), anti-thyroglobulin antibody (TgAb), aspartate aminotransferase (AST), alanine aminotransferase (ALT), blood cell counts (leukocyte, neutrophil, and lymphocyte counts), the specific type of initial ATD, and the initial daily dose of ATDs. The primary outcome was relapse within one year after ATD withdrawal, defined as biochemically confirmed overt thyrotoxicosis meeting either of the following criteria: (i) TSH below the lower limit of the reference range together with FT4 and/or FT3 above the upper limit of the reference range; or (ii) recurrence of typical signs and symptoms of hyperthyroidism accompanied by re-initiation or dose escalation of ATD, or by receipt of radioactive iodine therapy or thyroidectomy as treatment for the recurrence. Biochemical relapse according to criterion (i) was confirmed by a single documented measurement meeting these thresholds; no repeat measurement or minimum interval between measurements was required.

### 2.4 Sample size and missing data handling

The overall dataset was randomly partitioned into a training set and a testing set using a 70%/30% split, with stratified division employed to ensure a balanced proportion of the outcome variable (relapse) between the two groups. Prior to model construction, missing data patterns and proportions were assessed for all candidate variables. Variables with more than 30% missing data were excluded from model construction. all candidate variables, including those with high missingness, are shown descriptively in **Table 1**. Patients in whom any of the five final predictors was missing could not be assigned a predicted probability and were therefore excluded from the ROC analysis at the model-evaluation stage. In the training set, for variables retained with lower levels of missingness, a mode imputation was first applied to categorical predictors and a k-nearest-neighbors imputation to continuous predictors as part of the preprocessing pipeline for LASSO regression. Subsequently, a multiple imputation strategy was adopted for the final set of predictors selected by LASSO and clinical judgement. The imputation was implemented using the mice (Multivariate Imputation by Chained Equations) package in R, generating 5 complete datasets with a maximum of 10 iterations, and the imputation model included the outcome variable and all candidate predictors to ensure its validity. The subsequent multivariable logistic regression was performed on these 5 multiply imputed datasets, and results were pooled according to Rubin’s rules.

**Table 1:** Baseline characteristic.

Relapse status				
Characteristic	Overall, N = 654^1^	No, N = 257^1^	Yes, N = 397^1^	p-value^2^
Gender				0.03
male	161 (24.6%)	51 (19.8%)	110 (27.7%)	
female	493 (75.4%)	206 (80.2%)	287 (72.3%)	
Age (years)	37.00 (26.00, 51.00)	48.00 (33.00, 55.00)	32.00 (22.00, 45.00)	<0.01
Time from onset to diagnosis (weeks)	8.00 (4.00, 24.00)	4.00 (3.00, 20.00)	8.00 (4.00, 24.00)	<0.01
Exophthalmos				0.39
no	347 (80.1%)	137 (82.5%)	210 (78.7%)	
yes	86 (19.9%)	29 (17.5%)	57 (21.3%)	
Goiter grade				<0.01
0	282 (43.4%)	148 (58.0%)	134 (33.9%)	
1	88 (13.5%)	49 (19.2%)	39 (9.9%)	
2	252 (38.8%)	56 (22.0%)	196 (49.6%)	
3	28 (4.3%)	2 (0.8%)	26 (6.6%)	
Thyroid volume (cm³)	21.76 (16.96, 28.83)	19.90 (14.00, 25.68)	23.44 (17.44, 30.63)	0.01
Lymphocyte count (×10^9^/L)	1.97 (1.60, 2.41)	1.85 (1.47, 2.20)	2.07 (1.67, 2.53)	<0.01
Neutrophil count (×10^9^/L)	2.91 (2.30, 3.83)	2.90 (2.36, 3.97)	2.92 (2.29, 3.72)	0.66
NLR	1.50 (1.11, 2.10)	1.60 (1.21, 2.25)	1.39 (1.05, 1.98)	<0.01
TSH baseline	0.01 (0.01, 0.01)	0.01 (0.01, 0.01)	0.01 (0.01, 0.01)	0.01
≤0.005	580 (89.5%)	220 (85.6%)	360 (92.1%)	
>0.005	68 (10.5%)	37 (14.4%)	31 (7.9%)	
FT4 baseline (pmol/L)	65.12 (43.06, 100.00)	51.50 (35.90, 76.97)	78.30 (51.85, 100.00)	<0.01
FT3 baseline (pmol/L)	26.49 (16.35, 39.23)	20.27 (13.47, 30.80)	31.70 (21.08, 43.21)	<0.01
TPOAb baseline (IU/mL)	168.15 (34.91, 387.08)	114.15 (19.14, 337.63)	213.15 (65.73, 403.98)	<0.01
TRAb baseline (IU/L)	14.86 (7.66, 24.87)	8.40 (5.01, 15.08)	21.02 (12.20, 30.00)	<0.01
TgAb baseline (IU/mL)	180.65 (24.44, 627.60)	219.10 (25.45, 473.15)	165.80 (23.25, 821.50)	0.78
Initial medication type				0.65
Propylthiouracil	19 (2.9%)	6 (2.3%)	13 (3.3%)	
Methimazole	635 (97.1%)	251 (97.7%)	384 (96.7%)	
Initial dose (mg/day)				<0.01
5	7 (1.1%)	5 (1.9%)	2 (0.5%)	
10	26 (4.0%)	14 (5.4%)	12 (3.0%)	
15	35 (5.4%)	22 (8.6%)	13 (3.3%)	
20	162 (24.8%)	72 (28.0%)	90 (22.7%)	
30	424 (64.8%)	144 (56.0%)	280 (70.5%)	

^1^ Continuous variables: normal distributed data shown as Mean ± SD; non-normal as Median (IQR). P-values: t-test for normal, Wilcoxon rank-sum test for non-normal, Chi-square test for categorical. Variables with >30% missing data were not included in model building and are shown for descriptive purposes only.

^2^ Pearson’s Chi-squared test; Wilcoxon rank sum test.

### 2.5 LASSO regression

To screen for robust predictors of GD relapse while avoiding overfitting, LASSO regression was applied within the R environment. The glmnet package was used to fit a LASSO logistic regression model (alpha = 1, with internal standardization disabled due to prior standardization), and the optimal penalty parameter lambda was selected via 10-fold cross-validation using deviance as the evaluation metric. The lambda.min value, corresponding to the minimum cross-validation deviance, was selected, and the variables with non-zero coefficients at this lambda value were retained as key features for predicting GD relapse.

### 2.6 Model development and statistical analysis

The variables identified by LASSO were incorporated into a multivariable binary logistic regression model within the imputed training set. For a clinically relevant variable such as goiter grade, the four ordinal categories (0, I, II, III) were consolidated into a binary variable (grade 0–I vs. grade II–III) to simplify the model and enhance its clinical applicability. The final model is presented by reporting the adjusted odds ratios (ORs) for each predictor, along with their 95% confidence intervals (CIs) and corresponding p-values.

### 2.7 Model performance and validation

The performance of the final logistic regression model was comprehensively assessed from three perspectives: discrimination, calibration, and clinical utility. Discrimination was evaluated using the receiver operating characteristic (ROC) curve, with the area under the curve (AUC) and its 95% CI calculated for both the training and testing sets to quantify the model’s ability to distinguish patients who relapsed from those who did not. Calibration, which reflects the agreement between model-predicted probabilities and observed outcomes, was assessed using a calibration plot, on which a Loess smooth curve was superimposed to visually inspect for any systematic deviation from ideal prediction. Clinical utility and net benefit were assessed through decision curve analysis (DCA), which reflects the practical value of the model across a range of clinically relevant threshold probabilities used to classify patients as high risk. Internal validation of the final model was performed by bootstrap resampling with 1000 replicates using the rms package in R (rms::validate), from which the optimism-corrected C-index (equivalent to the AUC) and the optimism-corrected calibration intercept and slope were derived.

### 2.8 Comparison with the GREAT score

To benchmark the performance of the newly constructed model, we compared it against the established Graves Recurrent Events After Therapy (GREAT) score. Based on the original algorithm proposed by Vos et al. (19), the GREAT score was calculated for each patient in the testing set as the sum of the following points: age <40 years, +1; FT4 ≥40 pmol/L, +1; TBII/TRAb <6 IU/L, 0 points, 6–19.9 IU/L, +1 point, and ≥20 IU/L, +2 points; and goiter grade 0–I, 0 points, goiter grade II–III, +2 points (total score range 0–6). Patients were also classified into GREAT risk classes I (0–1 points), II (2–3 points), and III (4–6 points). The discriminative performance of both our nomogram model and the GREAT score was evaluated by constructing their respective ROC curves within the same testing set. The AUCs of the two models were compared, and the DeLong test was employed to assess the statistical significance of the difference. A p-value <0.05 from the DeLong test was considered indicative of a statistically significant difference in model performance.

### 2.9 Nomogram

To facilitate the application of this model for individualized risk assessment in clinical practice, a visual nomogram was constructed based on the final logistic regression model. Using the rms package in R software, the regression coefficients of the independent predictors in the final model were rendered into a graphical scoring system. By summing the points corresponding to a patient’s specific clinical characteristics, clinicians can directly map the total score to the predicted probability of GD relapse after ATD discontinuation. Furthermore, an interactive online calculator was developed and deployed to provide instantaneous, individualized risk estimation at the point of care.

## 3 Results

### 3.1 Baseline characteristics

A total of 654 patients with GD who achieved remission after at least 12 months of ATD therapy and subsequently discontinued treatment were enrolled, of whom 257 maintain remission (non-relapse group) and 397 experience relapse within one year after ATD discontinuation (relapse group). Baseline demographic, clinical, and laboratory characteristics stratified by relapse status are summarized in **Table 1**. The overall median age is 37.0 years (interquartile range [IQR], 26.00–51.00). Patients in the relapse group are significantly younger than those in the non-relapse group (median 32.0 [IQR, 22.00–45.00] vs. 48.0 [33.00–55.00] years; p < 0.01), and the proportion of females is lower (72.3% vs. 80.2%; p = 0.03). The relapse group exhibits a significantly higher proportion of goiter grade 2–3 (56.2% vs. 22.8%; p < 0.01), a larger thyroid volume (median 23.44 vs. 19.90 cm³; p = 0.01), and a longer interval from symptom onset to diagnosis (median 8.00 vs. 4.00 weeks; p < 0.01). With respect to laboratory parameters, baseline thyroid hormone and antibody levels differ markedly between the two groups. Relapse patients demonstrate significantly elevated median concentrations of FT4 (78.30 vs. 51.50 pmol/L; p < 0.01), FT3 (31.70 vs. 20.27 pmol/L; p < 0.01), TRAb (21.02 vs. 8.40 IU/L; p < 0.01), and TPOAb (213.15 vs. 114.15 IU/mL; p < 0.01). In addition, lymphocyte count, neutrophil-to-lymphocyte ratio (NLR), TSH, and the initial ATD dose are also significantly different between the groups (all p < 0.05). In contrast, the presence of ophthalmopathy, neutrophil count, TgAb levels, and the type of initial ATD (propylthiouracil vs. methimazole) do not show statistically significant differences between the two groups (all p > 0.05).

### 3.2 Predictor selection by LASSO regression

To prevent model overfitting and identify the most robust predictors from the candidate variable pool, LASSO regression with 10-fold cross-validation is performed on the training set. The optimal regularization parameter (lambda) is chosen based on lambda.min. As shown in **Figure 1**, this step retains eight predictors with non-zero coefficients: sex, age, goiter grade (dichotomized as grade 0–1 vs. grade 2–3), baseline FT4, TSH, initial drug type, time from onset to diagnosis, and baseline TRAb. These variables are subsequently entered into the multivariable logistic regression model. We also examined whether the baseline FT3/FT4 ratio added incremental information beyond the final model. The ratio was higher in patients who subsequently relapsed (median 0.427, IQR 0.373–0.477) than in those who did not (median 0.386, IQR 0.340–0.453; Mann–Whitney p < 0.001), but it was not retained by LASSO (coefficient 0) and adding it to the final model did not improve discrimination (delta AUC = -0.014, 95% CI: -0.043 to 0.002; the 95% CI included 0); its adjusted odds ratio in the extended model was 1.70 (95% CI: 0.12–24.20; p = 0.69).

**Figure 1:**
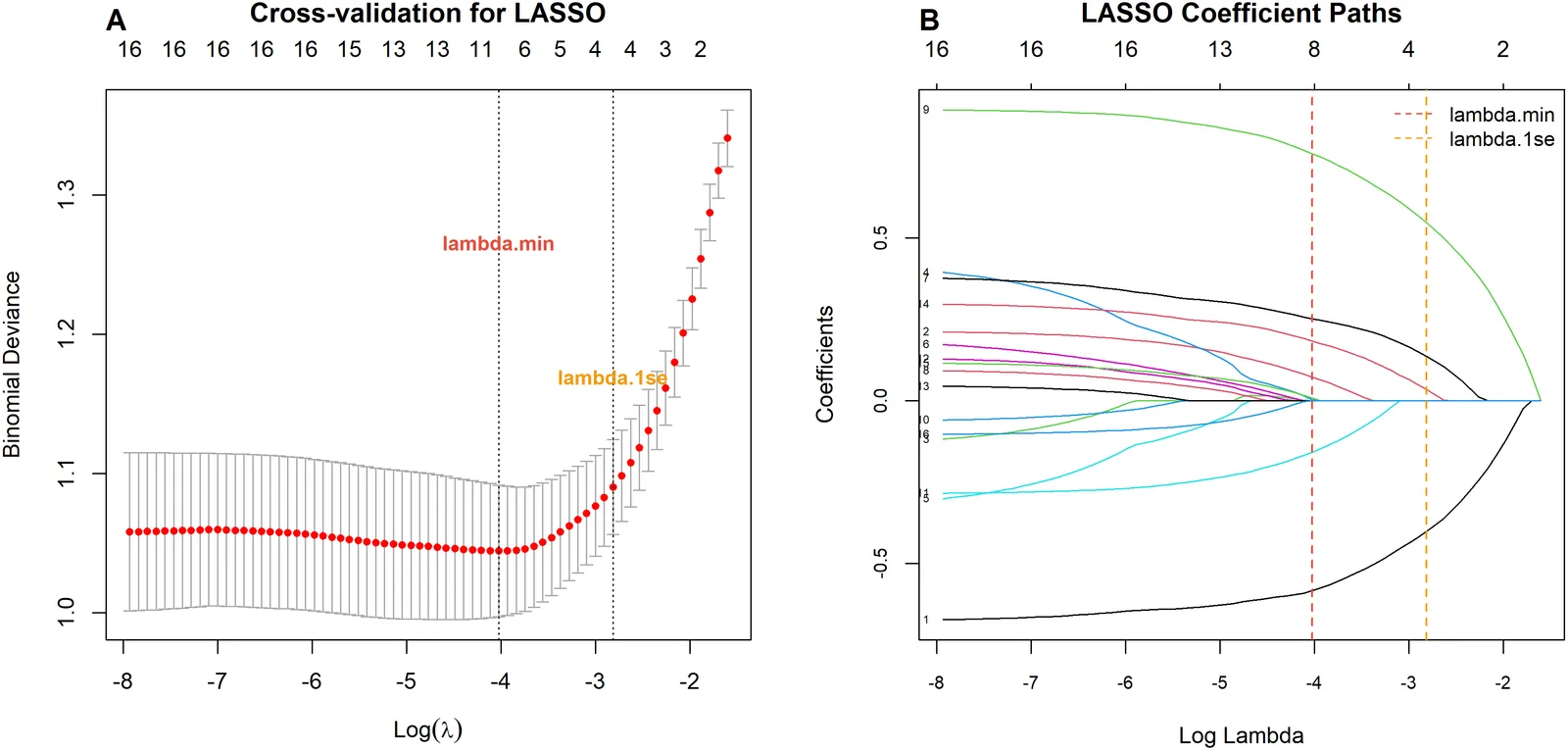
LASSO regression for predictor selection. **(A)** Ten-fold cross-validation error curve plotted against the logarithm of the lambda (λ) sequence. The left vertical dashed line indicates lambda.min, and the right vertical dashed line indicates lambda.1se. **(B)** Coefficient paths of the candidate predictors across the log(λ) sequence. Eight predictors retained non-zero coefficients at lambda.min: sex, age, goiter grade (dichotomized as grade 0–1 vs. grade 2–3), baseline free thyroxine (FT4), thyroid-stimulating hormone (TSH), initial drug type, time from symptom onset to diagnosis, and baseline thyrotropin receptor antibody (TRAb).

### 3.3 Independent risk factors determined by multivariable logistic regression

The results of the multivariable logistic regression analysis on the multiply imputed datasets, after adjusting for mutual confounding, are presented in **Table 2**. Five baseline characteristics are identified as independent predictors of GD relapse after ATD discontinuation. Compared with males, female sex is associated with a significantly lower risk of relapse (adjusted OR 0.49, 95% CI 0.27–0.87; p = 0.02). Each one-year increase in age exerts a protective effect (adjusted OR 0.96, 95% CI 0.94–0.97; p < 0.01). Conversely, the presence of goiter grade 2–3, relative to goiter grade 0–1, is a significant risk factor (adjusted OR 1.88, 95% CI 1.13–3.11; p = 0.01). Elevated baseline FT4 level (adjusted OR 1.01, 95% CI 1.00–1.02; p = 0.01) and higher baseline TRAb titer (adjusted OR 1.08, 95% CI 1.06–1.11; p < 0.01) are both independently associated with increased relapse risk. Notably, the other variables included in the model, including time to diagnosis, TSH, and initial drug type, do not retain independent statistical significance (all p > 0.05).

**Table 2:** Independent predictors of Graves’ disease relapse within one year after antithyroid drug withdrawal determined by multivariable logistic regression.

Variable^1^	Adjusted OR^2^	95% CI^2^	p-value
Gender			
male	**—**	**—**	
female	**0.49**	**0.27, 0.87**	**0.02**
Age (years)	**0.96**	**0.94, 0.97**	**<0.01**
Time from onset to diagnosis (weeks)	1.01	1.00, 1.02	0.09
Goiter grade			
Grade 0–1	**—**	**—**	
Grade 2–3	**1.88**	**1.13, 3.11**	**0.01**
FT4 (pmol/L)	**1.01**	**1.00, 1.02**	**0.01**
TRAb (IU/L)	**1.08**	**1.06, 1.11**	**<0.01**
TSH (mIU/L)	4.79	0.48, 47.80	0.56
Initial medication type			
Propylthiouracil	—	—	
Methimazole	0.48	0.12, 1.80	0.28

^1^ Variables in **bold** were retained in the final prediction model (overall p < 0.05).

^2^ OR, Odds Ratio, CI, Confidence Interval.

### 3.4 Model performance and validation

The final logistic model demonstrates favorable discriminative ability in both the training and testing sets. The AUC is 0.82 (95% CI: 0.78–0.86) in the training set and 0.84 (95% CI: 0.79–0.90) in the testing set (**Figure 2A**). In the testing set, 10 of the 198 patients (5.1%) had incomplete data for at least one of the five model variables (TRAb, n = 6; FT4, n = 2; goiter grade, n = 2; one patient was missing both TRAb and FT4) and could not be assigned a predicted probability; both the ROC curve of the model and the comparison with the GREAT score were consequently based on the same 188 complete cases. The relapse rate among these 10 excluded patients (7/10, 70.0%) did not differ significantly from that among the 188 included patients (113/188, 60.1%; chi-square P = 0.77; Fisher exact P = 0.74), suggesting no substantial systematic difference, although the small number of excluded cases limits the power of this comparison. The performance in the testing set is comparable to, and slightly better than, that in the training set. The calibration curves indicate satisfactory agreement between the model-predicted probabilities and the actual observed proportions of relapse in both datasets (**Figure 2B**). Bootstrap internal validation with 1000 replicates showed an optimism of 0.0075 and an optimism-corrected C-index of 0.82 (range across the five imputed datasets, 0.81–0.82). In the testing set, logistic calibration regression yielded a calibration intercept of -0.17 (95% CI: -0.53 to 0.18) and a calibration slope of 1.09 (95% CI: 0.77–1.42), indicating agreement between predicted and observed risks without systematic over- or under-estimation. Although minor fluctuations are observed in the lower-to-moderate probability range of the testing set, possibly attributable to reduced sample sizes within individual calibration bins, the smoothed calibration curves for both sets consistently remain close to the ideal 45-degree reference line, suggesting acceptable overall calibration. Decision curve analysis (**Figure 2C**) demonstrates that applying the model to guide clinical management provides a positive net benefit over the treat-all and treat-none strategies across a range of clinically reasonable threshold probabilities in both the training and testing sets. These results support the practical clinical utility of the model for risk-stratified decision-making.

**Figure 2:**
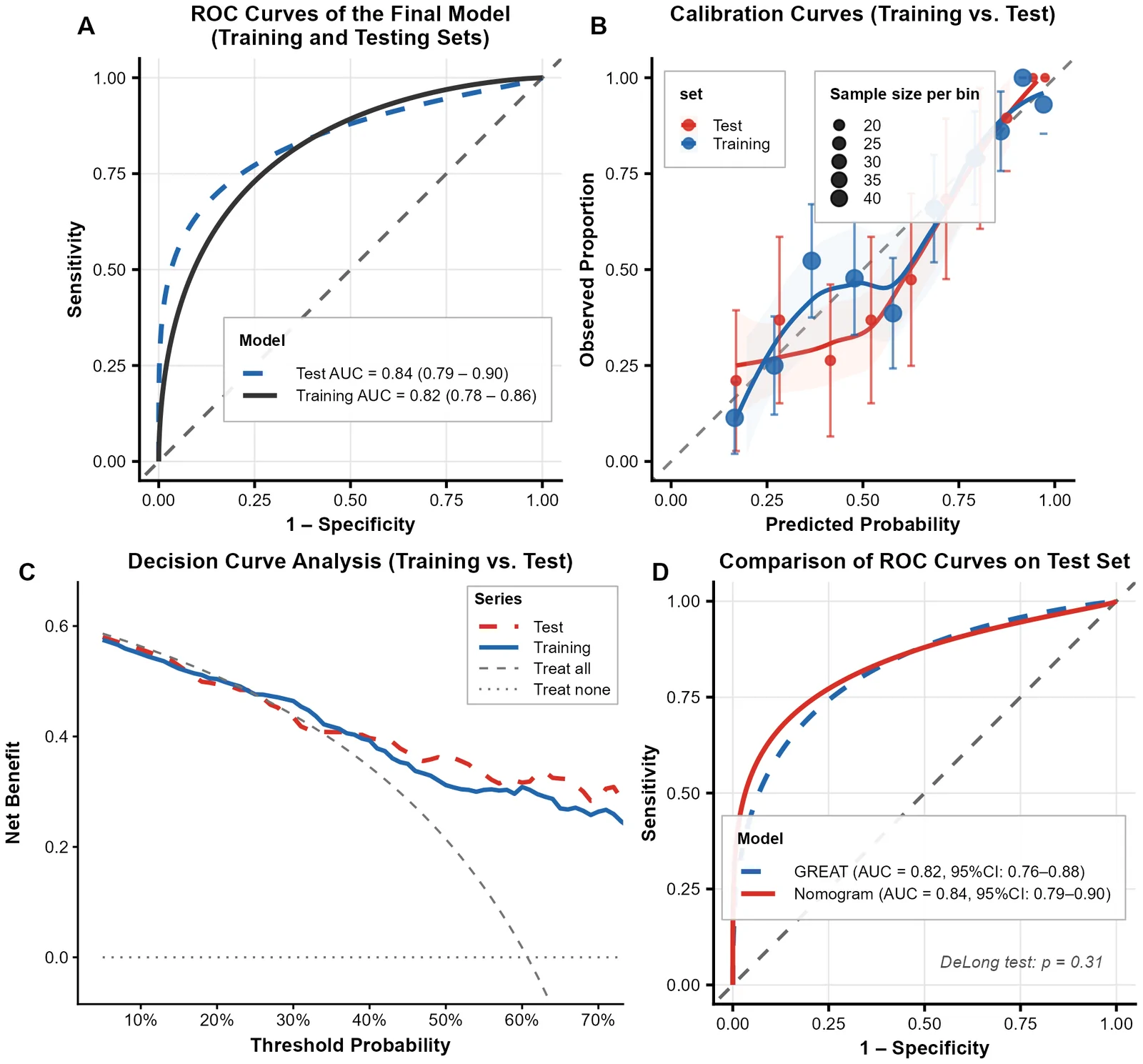
Performance evaluation and comparison of the final prediction model. **(A)** Receiver operating characteristic (ROC) curves of the final logistic model in the training set and testing set. The diagonal dashed line represents the reference line of no discrimination. **(B)** Calibration curves comparing predicted probabilities with observed proportions of relapse in the training and testing sets. The diagonal dashed line indicates perfect calibration. **(C)** Decision curve analysis showing net benefit across threshold probabilities in the training and testing sets. The gray dashed line represents the treat-all strategy, and the black dashed line represents the treat-none strategy, and the colored lines represent the prediction model. **(D)** Comparison of ROC curves between the new nomogram model and the Graves Recurrent Events After Therapy (GREAT) score in the testing set. The area under the ROC curve (AUC) was 0.84 (95% CI: 0.79–0.90) for the new model and 0.82 (95% CI: 0.76–0.88) for the GREAT score computed with the original Vos algorithm. The difference between the two AUCs was not statistically significant (DeLong test, P = 0.31). ROC, receiver operating characteristic; AUC, area under the curve; GREAT, Graves Recurrent Events After Therapy; CI, confidence interval.

### 3.5 Comparison of predictive performance between the new model and the GREAT score

In a direct comparison on the same independent testing set, the new nomogram model and the GREAT score showed comparable discrimination. The AUC was 0.84 (95% CI: 0.79–0.90) for the new model and 0.82 (95% CI: 0.76–0.88) for the GREAT score computed with the original Vos algorithm (**Figure 2D**). The difference between the two AUCs was not statistically significant (DeLong test, p = 0.31; mean difference in AUC 0.018, 95% CI −0.019 to 0.055 by bootstrap), indicating comparable discriminative performance in this Chinese patient cohort. In contrast to the GREAT score, which assigns points using fixed cut-offs for FT4 and TBII, our model uses both variables as continuous measures and returns an individualized probability of relapse rather than a discrete score. When the patients in the testing set were classified according to the original GREAT risk classes, the observed relapse rate increased across classes: 20.0% (6/30) in class I, 43.3% (29/67) in class II, and 85.7% (78/91) in class III, in the same direction as the gradient reported in the original derivation cohort (19).

### 3.6 Risk stratification and threshold performance

Because the final model returns a continuous probability rather than a discrete score, we examined its ability to stratify patients by predicted risk in the testing set. When patients were grouped into quartiles of predicted probability, the observed relapse rate increased monotonically from 23.4% (11/47) in Q1 to 42.6% (20/47) in Q2, 76.6% (36/47) in Q3, and 97.9% (46/47) in Q4 (Cochran-Armitage test for trend p < 0.001). Applying the same cut-offs used in the decision curve analysis, a predicted probability of at least 20% identified 96.5% (95% CI: 91.2–99.0) of the patients who relapsed, with a specificity of 14.7% (7.6–24.7), a positive predictive value of 63.0% (55.3–70.2), and a negative predictive value of 73.3% (44.9–92.2); at a cut-off of at least 40%, the corresponding values were 90.3% (83.2–95.0), 45.3% (33.8–57.3), 71.3% (63.2–78.6), and 75.6% (60.5–87.1), respectively (**Figure 3**).

**Figure 3:**
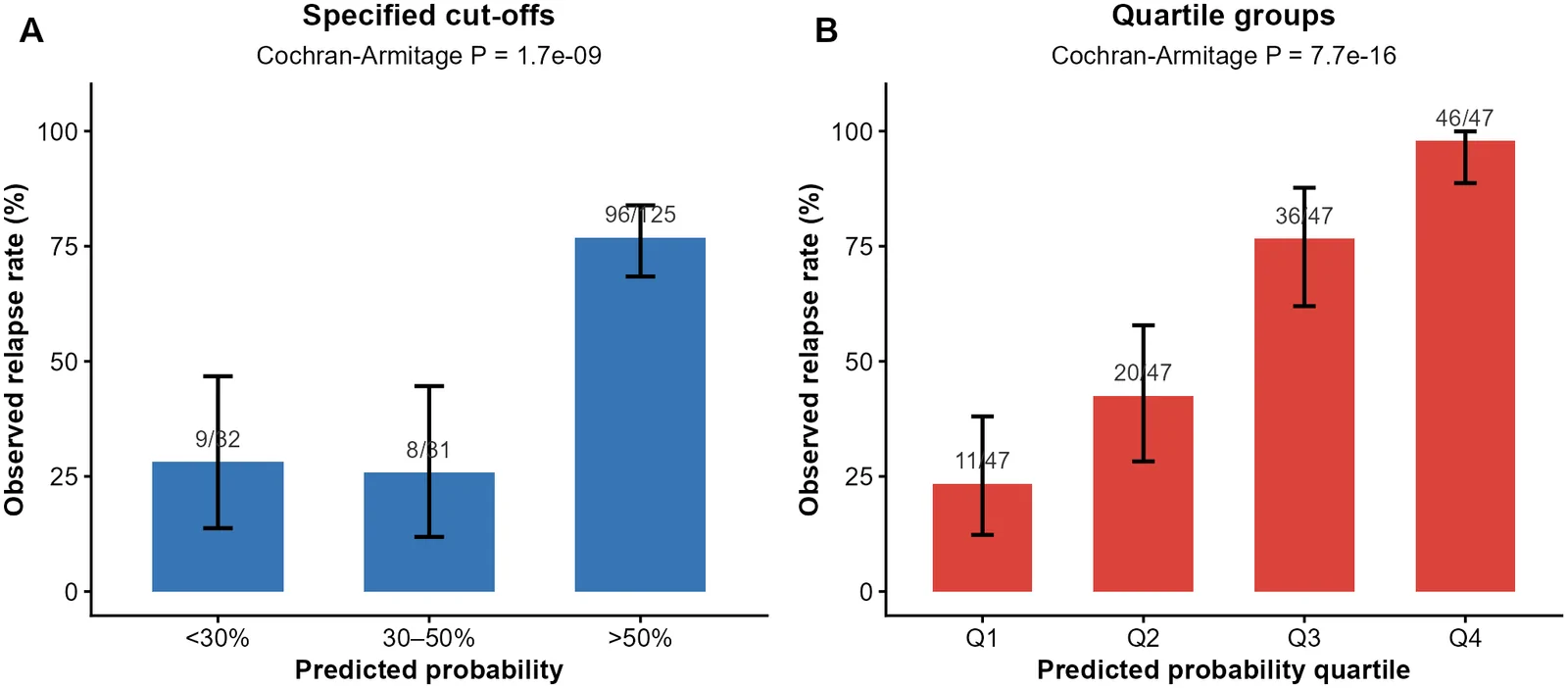
Risk stratification by the final model in the testing set. **(A)** Observed relapse rate according to pre-specified predicted-risk groups (<30%, 30–50%, and >50%). **(B)** Observed relapse rate according to quartiles of predicted probability. Error bars represent 95% confidence intervals, and the fractions above each bar indicate the number of patients who relapsed relative to the total number of patients in that group.

### 3.7 Construction of a nomogram for clinical application

To facilitate individualized risk assessment at the point of care, a user-friendly nomogram is constructed based on the final logistic regression model (**Figure 4**). This nomogram integrates the five independent predictors—sex, age, goiter grade, baseline FT4, and baseline TRAb—into a comprehensive scoring system. Clinicians can sum the points corresponding to a patient’s individual characteristics and directly map the total score to the predicted probability of GD relapse within one year after ATD discontinuation, thereby enabling quantitative, personalized risk communication and shared clinical decision-making. An interactive online version of this nomogram was made publicly available to streamline clinical use and enhance accessibility. (https://drh923936332.shinyapps.io/thyroid-relapse-nomogram/).

**Figure 4:**
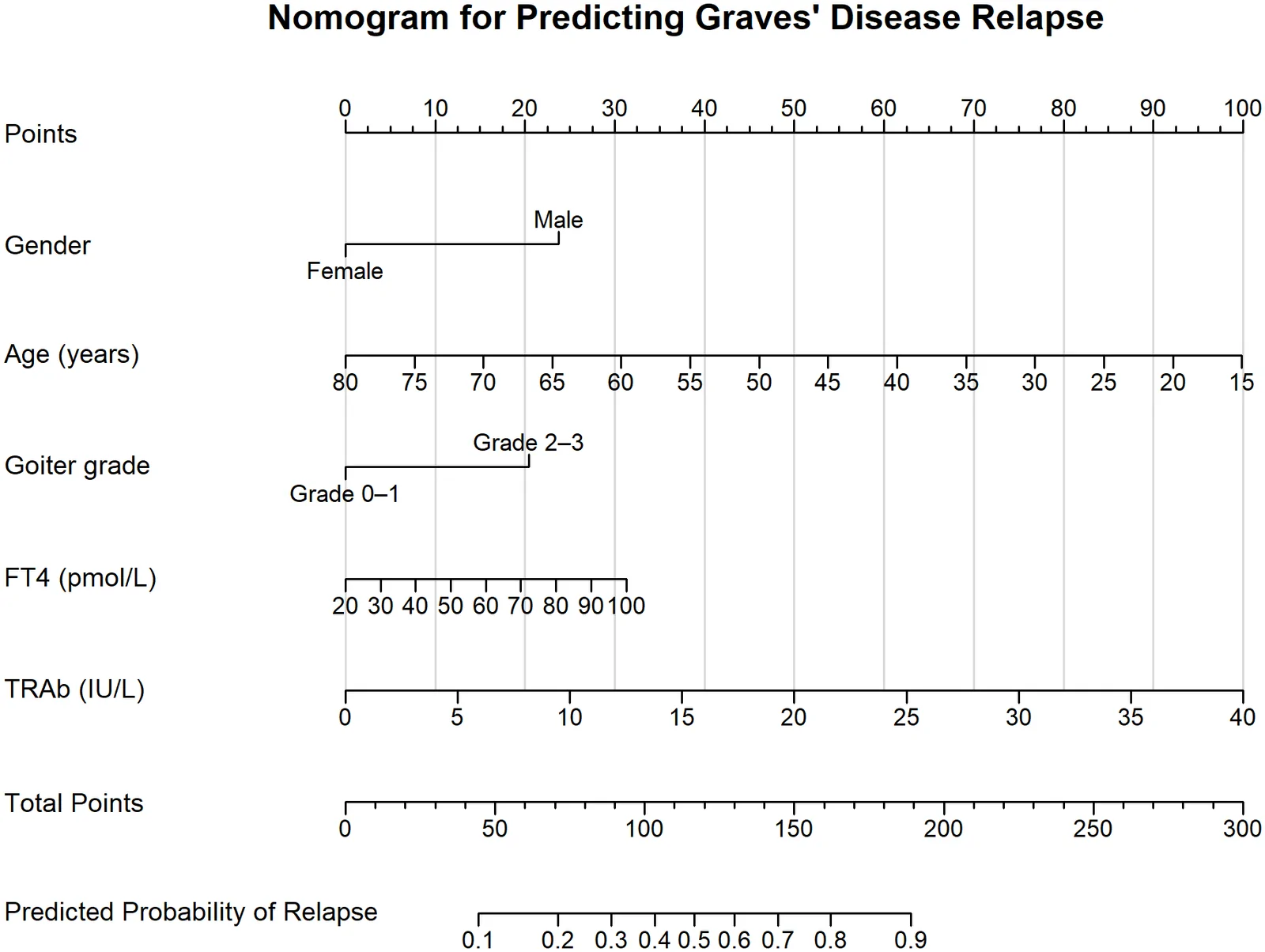
Nomogram for predicting the probability of Graves’ disease relapse after antithyroid drug withdrawal. The nomogram integrates five independent baseline predictors: sex, age, goiter grade, baseline free thyroxine (FT4), and baseline thyrotropin receptor antibody (TRAb). To obtain the predicted probability, locate the patient’s value on each variable axis, draw a line upward to the Points axis to determine the corresponding score, sum the points for all variables, and locate the total points on the Total Points axis to draw a line down to the Predicted Probability axis. FT4, free thyroxine; TRAb, thyrotropin receptor antibody.

## 4 Discussion

The management of GD involves complex decision-making ATDs, RAI, and surgery, and the high relapse rate after ATD discontinuation represents a central concern influencing the treatment preferences of both patients and clinicians (8, 9). Although ATDs have become the first-line therapeutic option worldwide (7, 10), the prediction of individualized relapse risk remains a critical unmet need, as personalized risk assessment is rarely discussed during clinical encounters despite its pivotal role in shared decision-making (17, 18). Against this background, we aimed to identify baseline predictors of GD relapse within one year after ATD withdrawal and to construct a practical, accessible prediction model. This single-center retrospective analysis enrolled 654 patients with GD who achieved remission after at least 12 months of ATD therapy and subsequently discontinued treatment, and identified five independent risk factors, namely younger age, male sex, goiter grade 2–3, higher baseline FT4, and elevated TRAb levels, which were integrated into a LASSO regression-based logistic regression nomogram. The model demonstrated robust discriminative performance in an independent testing set, with an AUC of 0.84, showing discrimination comparable to that of the established GREAT score (AUC 0.84 vs. 0.82; DeLong P = 0.31) in this Chinese cohort. This concise five-variable model relies exclusively on demographic, clinical, and routine laboratory parameters, providing a practical, user-friendly tool for individualized risk assessment at the critical juncture of initial treatment decision-making.

Our findings confirm and extend the existing literature on the prediction of GD relapse. Consistent with previous reports, younger age, higher baseline FT4, and elevated TRAb titer are reaffirmed as robust independent predictors of relapse (19–22). The association between young age and a poorer prognosis is well documented and may reflect a more vigorous or dysregulated autoimmune response, making the spontaneous restoration of immune tolerance more difficult to achieve during ATD therapy (20, 23, 24). Similarly, an elevated FT4 level signifies a greater hyperthyroid burden and is mechanistically linked to the overexpression of B-cell activating factor (BAFF), which may perpetuate active autoimmunity and predispose to relapse (25). As the pathogenic hallmark of GD, TRAb directly drives thyrocyte stimulation; its persistently high titer implies ongoing immune activity that is likely to resurge after drug discontinuation (4, 22, 26). The independent predictive value of goiter grade 2–3, relative to a smaller gland, is consistent with numerous studies (19, 21, 27, 28) and likely reflects both a state of prolonged thyroid stimulation and the inherently greater vascularity of a larger goiter, which provides a larger substrate for recurrent hyperthyroidism (3). Male sex emerged as a risk factor in our cohort, and although not confirmed in all investigations (20, 29), it is supported by a series of large-scale studies and meta-analyses demonstrating that male GD patients exhibit lower remission rates, larger goiters at initial diagnosis, and possibly a stronger genetic autoimmune predisposition, collectively contributing to a more refractory disease phenotype (11, 24, 30, 31). In our univariate analysis, a longer time from symptom onset to diagnosis was also associated with relapse (**Table 1**), suggesting that a more prolonged interval of untreated hyperthyroidism before diagnosis may reflect more advanced disease; however, this signal was attenuated in the multivariable context, most likely due to confounding introduced by the severity markers already included. Of note, the median NLR was lower in the relapse group than in the non-relapse group (1.39 vs. 1.60; **Table 1**), which is opposite to the direction reported in some previous studies; NLR was not retained in the final model, and the clinical significance of this univariate finding therefore remains uncertain. A recent study in pediatric autoimmune hyperthyroidism reported that a higher baseline FT3/FT4 ratio predicted relapse after ATD withdrawal (54). In our adult cohort the ratio was likewise higher in patients who subsequently relapsed (median 0.427 vs. 0.386; p < 0.001), but it was not selected by LASSO, and adding it to the final model did not improve discrimination (delta AUC = -0.014, 95% CI: -0.043 to 0.002). This discrepancy may reflect differences in population (pediatric versus adult) and in assay platforms and reference ranges; in addition, FT4 values in our cohort were truncated at the upper reporting limit of 100 pmol/L, which compresses the ratio.

GO has been identified as a predictor in previous studies (20, 21, 27). The absence of an independent GO effect in our primary analysis may be attributable to the relatively mild severity of ophthalmopathy in our cohort, as patients with severe, active GO typically seek other definitive treatments earlier and are less likely to undergo prolonged ATD monotherapy, thereby introducing selection bias. The substantial heterogeneity among published cohorts is another major source of these discrepancies. These datasets differ considerably in sample size, regional iodine sufficiency (1, 32), ATD regimen (titration versus block-and-replace) (33, 34), treatment duration (35, 36), and the timing of risk factor assessment (at diagnosis versus at ATD discontinuation) (20, 37).

A key limitation of many existing studies, including our own, is the handling of missing data. For instance, the GREAT score used for comparison was originally developed using TRAb (specifically TBII) and goiter size, parameters that are not consistently available in all real-world settings (20). Our model relies predominantly on widely available laboratory tests (FT4, TRAb) and simple clinical assessments (age, sex, goiter palpation grade), which may partly circumvent this variability and thereby enhance its external applicability.

Our prediction model contributes to the growing armamentarium of GD risk stratification tools while offering distinct practical advantages. The GREAT score, based on age, serum FT4, TBII, and goiter size (20), along with its genetic extension (GREAT+) (19), represents a seminal contribution that has shown an AUC of approximately 0.70 with favorable calibration in both original and external validation cohorts (20, 38). In our testing set, our model showed discrimination comparable to that of the GREAT score (AUC 0.84 vs. 0.82, DeLong p = 0.31); however, because the model returns a continuous probability rather than a summed score, it stratifies patients across the full range of predicted risk, with observed relapse rates rising monotonically from 23.4% in the lowest quartile to 97.9% in the highest. Other tools, such as the nomogram by Baek et al. that integrates thyroid-stimulating immunoglobulin (TSI) and TBII (39) and the Clinical Severity Score (CSS) based on the Merseburg triad (40), have yielded AUCs in the range of 0.60–0.67. More recently, a risk scoring system that integrated shear wave elastography with routine clinical variables reported an AUC of 0.91 in a prospective cohort (52), although elastography is not universally available and requires dedicated equipment and expertise. A notable feature of our model is that it achieves performance comparable to these published instruments without incorporating costly or less accessible biomarkers, such as genetic polymorphisms (19), TSI bioassays (39), or artificial neural network analyses of subtle clinical symptoms (41). Its variables, namely age, sex, goiter palpation, FT4, and TRAb, are obtainable through standard initial workup in nearly all clinical settings, including resource-limited environments. This parsimony enhances the potential for direct translation into clinical practice, avoiding the delays and costs of specialized testing while facilitating personalized, evidence-based discussions between patients and clinicians at the point of care.

In the contemporary era of precision medicine, a quantified estimate of individualized relapse risk is essential to move beyond a generic statistic such as an approximately 50% relapse rate and to empower truly informed shared decision-making (17). Our nomogram serves exactly this purpose by translating a patient’s individual risk factor profile into a concrete probability of relapse within one year after ATD discontinuation. This allows clinicians, for example, to recommend ATDs as first-line therapy with greater confidence for patients with a low predicted risk while informing high-risk patients earlier about the advantages of definitive options such as RAI or thyroidectomy (8, 9). It must be acknowledged, however, that despite its favorable discrimination, our model remains imperfect in ruling out all relapses, reflecting the current state of the art in the field. In practical terms, a predicted probability of relapse below 20% supports ATD withdrawal with routine follow-up, whereas a predicted probability of 20–40% warrants intensified monitoring of thyroid function after withdrawal; above 40%, the balance of benefit shifts towards discussing definitive treatment with RAI or surgery. These thresholds are consistent with the relapse rate observed in our cohort (60.7%) and with the approximately 50% relapse rate reported after ATD withdrawal in the literature (53). Active exploration for new markers to improve predictive accuracy is underway. Investigators have explored functional antibody assays (e.g., bioassays of thyroid-stimulating antibody [TSAb]) (42), long non-coding RNA and microRNA expression profiles (43, 44), human leukocyte antigen (HLA) haplotypes (19), CTLA-4 gene polymorphisms (45, 46), serum metabolomics (47), and even complex mathematical modeling (48). Recently, artificial intelligence (AI) methods are being applied to discern subtle nonlinear interactions within routine clinical data that may be missed by conventional logistic regression (49). Future research must focus on external validation of our model across diverse populations and, critically, on the integration of multi-omics data using these AI-driven approaches to build next-generation predictive tools with high accuracy and clinical translatability (50).

Several methodological limitations of this study warrant careful consideration. First, its retrospective observational design inherently precludes causal inference, and our findings remain susceptible to bias from unrecorded confounders. Although we performed multiple imputation for missing data, the potential for indication confounding, whereby clinicians’ perception of disease severity influences both initial treatment intensity and the decision to discontinue ATDs, can never be fully eliminated. Second, smoking history, a well-recognized environmental trigger for GD onset that is also associated with a higher relapse risk (21) and a limitation shared by other Asian cohorts (14, 51), was not captured in the retrospective chart review; similarly, precise ultrasonographic thyroid volume measurements and TRAb levels at the end of treatment were unavailable, restricting the scope of our analysis and model development. Importantly, the TRAb assay used is a competitive binding assay (TBII), and we were unable to assess the incremental value of TSAb measurement. Third, although the sample size is considerable, it remains insufficient to robustly investigate potential interaction effects or to provide an independent external validation cohort beyond the random split method. The cohort consisted exclusively of Chinese Han patients from an iodine-sufficient region, which may limit the direct generalizability of our findings to other racial, ethnic, or iodine-deficient populations. Fourth, we did not account for comorbidity burden, concomitant medications (e.g., amiodarone, lithium), or post-withdrawal stressors, which are known modulators of thyroid autoimmunity and relapse risk. Fifth, because relapse events were ascertained from retrospective charts, we were unable to stratify relapse further according to the management subsequently received (re-initiation or escalation of ATD therapy versus definitive treatment with radioactive iodine or surgery). In addition, the relapse rate in our cohort (60.7%) is higher than the approximately 50% generally reported after ATD withdrawal, which probably reflects three factors: remission after at least 12 months of standard ATD therapy with confirmed biochemical stability was required before withdrawal, so the study population differs from cohorts with shorter or less strictly defined treatment courses; all patients were recruited at a single center in an iodine-sufficient region; and our outcome definition captured relapse managed by re-initiation or escalation of ATD therapy or by definitive treatment in addition to biochemically confirmed thyrotoxicosis, thereby capturing events that stricter definitions would miss (53). These limitations are partly offset by several methodological strengths: predictors were selected by LASSO regression with 10-fold cross-validation, missing data were handled by multiple imputation, and model performance was internally validated by bootstrap resampling (B = 1000), which showed only a small optimism (0.0075) and an optimism-corrected C-index of 0.82, with calibration intercept and slope close to their ideal values. Nevertheless, these estimates derive from a single-center retrospective cohort, and external validation in independent, multi-center populations is required before the nomogram can be recommended for routine clinical use.

## 5 Conclusion

In summary, we confirm that younger age, male sex, goiter grade 2–3, higher baseline FT4, and elevated TRAb levels are independent risk factors for GD relapse within one year after ATD discontinuation. Based on these five routinely available variables, we developed a nomogram and demonstrated favorable internal discrimination and calibration. In our testing set, discrimination was comparable to that of the existing GREAT score, while the model provided a graded, individualized estimate of relapse risk in a Chinese population. However, in the absence of external validation, these performance estimates may be optimistic, and the model should not yet be considered suitable for widespread clinical use. It may serve as a foundation for further prospective, multi-center validation studies that are necessary before any clinical implementation. Although its predictive accuracy, like that of all existing models, remains imperfect, this work represents a step toward moving from generic risk discussions to more structured, individualized risk estimation, provided that its transportability is confirmed in diverse populations and settings. Future studies are crucial for external validation, integration of dynamic on-treatment variables and new biomarkers, and exploration of advanced analytical approaches to build next-generation prognostic algorithms for GD.

## Data Availability

The raw data supporting the conclusions of this article will be made available by the authors, without undue reservation.
